# Current News

**Published:** 1904-12

**Authors:** 


### Current News.

### BANQUET TO DR. C. T. STOCKWELL.

Dr. Chester Twitchell Stockwell was the recipient last evening of such recognition of notable service as comes but rarely to a man while he is yet alive. Sixty-five friends of many professions, dentistry predominating, tendered him a complimentary banquet at the Massasoit House, of which the features, aside from the bountiful feast, were a preliminary reception and after-dinner speaking, which set forth in terms of fine eulogy the feeling of the company towards its honored guest.

The dinner was given in recognition of the great service done by Dr. Stockwell to dentistry as a science in bringing clearly before the world the germ theory of dental decay, which since his epoch-making paper has become everywhere recognized as the truth, upsetting and forever doing away with the old theory of acids as the cause of destruction. This paper preceded the notable experiments made by Dr. Miller, of Berlin, which demonstrated the truth of Dr. Stockwell's position. It was also in recognition of the many years of faithful and helpful work and counsel among the dentists of the Connecticut Valley. They have been talking for several years of giving him this dinner, and the further testimonial of books, and the convenient time had now come.

The early part of the evening was given over to a reception in the upper parlor of the hotel. At the right of Dr. Stockwell in the receiving party stood Joel H. Hendrick, and at the left Charles

Goodrich Whiting, both intimate friends of the doctor for many years. Other members of the party were Dr. Herbert Stockwell and Professor A. E. Dolbear, of Tufts College. After an hour spent in kindly greeting and reunion, the gathering repaired to the dining-room, where a substantial repast was enjoyed, enlivened by selections by the Home City Quartette and recitations by C. B. Richardson. The tables were arranged in the form of a U, and were radiant with chrysanthemums, contributed by Mrs. Stockwell and Dr. Stockwell's daughter. Autumn leaves and palms supplied further decorations.

The toastmaster for the speaking was Dr. Newton Morgan, of this city, at whose right sat Dr. Stockwell. As a prelude, Dr. George A. Maxwell, of Holyoke, read specimens of the letters of regret received from those of the one hundred and fifty invited guests who could not attend. All testified to the great esteem in which Dr. Stockwell was held throughout this and other countries. The first speaker was Dr. Andrew J. Flanagan, of this city.

Dr. A. J. Flanagan gave a clear history of the remarkable pioneer work which was done by Dr. Stockwell in assisting to establish the bacteriological theory of the decay of teeth. He called attention, in opening, to the great revolution in science and medicine during the past twenty-five years. Three immortal names stand as a synonym of what this revolution implies—Tyndall, Pasteur, and Koch. The germ theory of disease may be traced back with more or less distinctness for two hundred years before the days of Pasteur, but in a practical way the world is more largely indebted to Pasteur than any man who lived during the nineteenth century.

Professor A. E. Dolbear, of Tufts College, presented a view of Dr. Stockwell as one of the philosophers of science. The scientific students discovered and proclaimed facts, and then it was the work of the philosopher, in the broader view, to correlate these facts, and deduce from them their higher and spiritual significance. It is in this class, which many thinkers regard as the higher, that Dr. Stockwell is to be placed. From his close study of science he has drawn inspiration for faith in the ultimate excellence. Dr. Stockwell's philosophical writing, he said, was on a plane of its own because of his trust that the universe was built by one who knew how to do it, and his feeling that he did not need to worry about looking after it. All kinds of knowledge seemed welcome to him. He welcomed the truth, he believed that goodness would triumph. His work has been, then, the raising of mere fact to ideal planes, by deductions of a supreme purpose of good in all manifestations of life.

Charles Goodrich Whiting said that he should speak of the intimate qualities that had made Dr. Stockwell the man he was. His studies in science had developed a close affection for Nature, and it was from her mother-breasts of exhaustless nourishment that he had drawn his interior life. The brief remarks were closed by the familiar lines from Wordsworth's "Tintern Abbey," as expressing the character of Stockwell:

> For I have learned
> To look on Nature, not as in the hour
> Of thoughtless youth, but hearing oftentimes
> The still, sad music of humanity,
> Not harsh or grating, though of ample power
> To chasten and subdue. And I have felt
> A presence that disturbs me with the joy
> Of elevated thoughts; a sense sublime
> Of something far more deeply interfused,
> Whose dwelling is the light of setting suns,
> And the round ocean, and the living air,
> And the blue sky, and in the mind of man;
> A motion and a spirit, that impels
> All thinking things, all objects of all thought,
> And rolls through all things.
> Therefore am I still
> A lover of the meadows and the woods
> And mountains, and of all that we behold
> From this green earth,—of all the mighty world
> Of eye and ear,—both what they half create
> And what perceive; well pleased to recognize
> In Nature and the language of the sense
> The anchor of my purest thought, the nurse,
> The guide, the guardian of my heart; and soul
> Of all my moral being.

Dr. John Dowsley, of Boston, president of the Massachusetts Board of Registration, said in the course of his remarks that during the recent general convention in St. Louis Dr. Stockwell was inquired for more than any other man. Dr. Waldo E. Boardman, of Boston, president of the National Dental Association; Dr. Edgar O. Kinsman, of Cambridge, president of the Massachusetts

Dental Society; Albion F. Bemis, of Clinton, Massachusetts State Senator; Dr. James McManus, of Hartford, president of the Northeastern Dental Society; and Dr. Maxfield, of Holyoke, were other speakers. Dr. Maxfield's words were in his happy vein, and as an appropriate climax for the evening's pleasure he presented Dr. Stockwell, in behalf of his friends of the dental profession, a beautiful bookcase containing the world's great thinkers' series,—Kant, Hegel, Plato, Aristotle, Spencer, Mill, and others, in large library octavo; a set of John Burroughs's works, a set of Thoreau, and others. When the programme had been completed, Dr. Morgan invited Dr. Stockwell to speak, if he should so desire.

Dr. Stockwell, evidently touched by the tributes and expressions of good will towards him, gave voice to his feelings in a short address. He said that the older he grew the more convinced he was that kindness and good will is the fundamental fact in human nature. The antagonism, the competition, the jealousies and afflictions of life sometimes lead us to feel that hate or, at least, indifference is the fundamental fact, but it is not so. These belong to the superficial side of life, they are temporary, transient, evanescent. As an example of this Dr. Stockwell related his experience, when, some thirty-five years ago, after he had begun his professional career in Des Moines, Iowa, his health failed. The generous and warm-hearted friendliness of the people of the town and the indorsement which they gave him on his leaving, of his record as a dentist and a citizen, he would never forget. It was no small consolation to reflect that if the shades of these older friends could be present and listen to the kind words of the evening, they might not, after all, regret their indorsement of him. In closing he thanked the company warmly for their kindness, and said, " I can think of no greater boon, as one approaches the evening hour of life, than to be assured that he shall live in the hearts of his fellows. That I conceive to be the most desirable mansion in our Father's house. With that boon to look forward to, I think I may say this is, if not the happiest, at least the most blessed hour of my life. With this inadequate expression of gratitude I bid you all good-night."

Singing of an appropriate arrangement of " Auld Lang Syne" was the closing feature.

The following were present at the dinner:

Senator Albion F. Bemis, of Clinton; R. M. Chase, of Bethel,

Vt.; Dr. H. E. Stockwell, of Stockbridge; Dr. Carl R. Lindstrom, of Lynn; Dr. Waldo E. Boardman, of Boston; Dr. Edward O. Kinsman, of Cambridge; Dr. J. N. Davenport, of Northampton; Dr. Civilion Fores, of Bridgeport, Conn.; Professor A. E. Dolbear, of Tufts College; Dr. A. G. Doane, of Northampton; Dr. Edward B. Griffith and Dr. Frederick Hindsley, of Bridgeport, Conn.; Dr. L. C. Taylor, of Hartford; Dr. F. D. Murlless, of Windsor Locks; Dr. R. H. Clark, of Northampton; Dr. C. S. Gates, of Amherst; Dr. P. T. Nichols, of Northampton; Dr. A. J. Nims, of Turners Falls; Dr. James McManus, of Hartford; Dr. F. Milton Smith, of New York; Dr. Clinton W. Strang, of Bridgeport, Conn.; Dr. George A. Maxfield, of Holyoke; Dr. John H. Dowsley, of Boston; Dr. E. E. Mitchell, of Haverhill; Dr. C. Frank Bliven, of Worcester, Mass.; Dr. Charles McManus, of Hartford; Dr. A. C. Fones, of Bridgeport, Conn.; Dr. Henry McManus, of Hartford, Conn.; Dr. A. J. Cutting, of Southington, Conn.; Dr. Frederic D. Murlless, Jr., of Hartford; Dr. Albert W. Crosby, of New London, Conn.; Dr. W. H. Rider; Dr. Edward B. Dickinson, of Amherst; Dr. P. W. Soule, of Monson; and Dr. J. Wesley Shaw, Dr. Cornelius S. Hurlbut, Samuel D. Sherwood, William F. Adams, A. M. Ross, Harold Ward, Dr. Andrew J. Flanagan, Samuel Bowles, Dr. Newton Morgan, Dr. C. T. Stockwell, Dr. C. Wesley Hale, H. B. Powers, Dr. H. C. Medicroft, Dr. R. A. Baldwin, Dr. James Martin, Dr. Walter W. Swazey, Dr. P. H. Derby, Dr. De Witt C. Shaw, Dr. P. J. Woodward, Dr. E. T. Sherman, Edward S. Hitchcock, Dr. D. Hurlbut Allis, Charles Goodrich Whiting, J. H. Hendrick, Charles H. Churchill, Dr. F. E. Hopkins, Francke W. Dickinson, all of this city.—*Springfield Daily Republican.*

---

### INSTITUTE OF DENTAL PEDAGOGICS.

The Institute of Dental Pedagogics will hold its annual meeting at Louisville, Ky., December 28, 29, and 30, 1904. This has come to be the most important gathering of the year, and no dental teacher or practitioner interested in dental education can afford to miss the session.

H. B. Tileston, *President.*
W. E. Willmott, *Secretary.*

### HARTFORD DENTAL SOCIETY.

At the annual meeting of the Hartford Dental Society, held October 10, 1904, the following officers were elected for the ensuing year:

President, Dr. G. M. Griswold; Vice-President, Dr. C. E. Barrett; Secretary, Dr. A. E. Cary; Treasurer, Dr. E. R. Whitford; Librarian and Curator, Dr. Henry Dryhurst; Historian, Dr. Henry McManus.

*Executive Committee.*—Dr. E. H. Munger, Chairman, Dr. W. S. Youngblood, Dr. A. A. Hunt.

A. E. Cary,
*Secretary.*

---

### THIRTY-SIXTH ANNIVERSARY MEETING OF THE FIRST DISTRICT DENTAL SOCIETY OF THE STATE OF NEW YORK.

The First District Dental Society will celebrate its thirty-sixth anniversary by two great meetings on the 12th and 13th of December. The essayists of these meetings will be Dr. G. V. Black, of Chicago, and Dr. E. K. Wedelstaedt, of St. Paul. Dr. Black will read an exhaustive *résumé* on the subject of "Extension for Prevention," illustrating his lecture with one hundred and twenty-five or more lantern-slide pictures. Dr. R. H. Hofheinz will open the discussion. On December 13 Dr. E. K. Wedelstaedt will read a paper on the "Packing of Gold in Approximo-Occlusal Cavities in Bicuspids and Molars," illustrating his method with the use of clay in large wooden models of teeth. The discussion of this paper will be opened by Dr. G. V. Black, and followed by Dr. R. Ottolengui and Dr. B. Holly Smith. This meeting will be held at the New York Academy of Medicine, 17 West Forty-third Street, at eight o'clock in the evening.

In addition to this meeting the Clinic Committee has arranged for a most interesting clinical exhibition to be held during the afternoons of these days, at the Grand Central Palace, Lexington Avenue and Forty-third Street. This programme comprises porcelain work, X-ray, different methods of packing gold, orthodontia, exhibits of different anatomical and histological specimens, etc.

Dr. Black will exhibit new instruments for testing the finger-power in the handling of operative instruments. Every facility will be afforded to enable the largest number to witness the demonstrations. The entire mornings of both days will be devoted to manufacturers' exhibits, which will be an interesting feature of the programme. All communications pertaining to clinics or exhibits should be addressed to the chairman of the Clinic Committee, Dr. S. L. Goldsmith, 129 East Sixty-ninth Street.

The following dental societies have been officially invited: The New York Institute of Stomatology, the New York Odontological Society, the New York Institute of Dental Techniques, the Second District Dental Society of the State of New York, the Central Dental Association of Northern New Jersey, the Hartford Dental Society, and the New Haven Dental Society.

A cordial invitation is extended to all members of the dental profession, and it is confidently expected that a large number of prominent dentists will be in attendance.

F. L. Fossume, *Chairman,*
A. M. Merritt,
A. G. Lansing,
*Executive Committee.*

---

### OHIO STATE DENTAL SOCIETY—THIRTY-NINTH ANNUAL MEETING.

Following is the programme of the thirty-ninth annual meeting of the Ohio State Dental Society, to be held at the Great Southern Hotel, Columbus, Ohio, December 6, 7, and 8, 1904:

### ESSAYS—TUESDAY.

"Personality," Bert E. Saunders, Elyria. Discussion, C. R. Butler, A. O. Ross.

"The Nerve Broach," W. I. Jones, Nelsonville. Discussion, H. J. Bosart, W. A. Holmes.

"Care of the Teeth of the Poor," W. T. Jackman, Cleveland. Discussion, C. I. Keely, W. G. Ebersole.

"The Dental Nurse," C. M. Wright, Cincinnati. Discussion, J. R. Callahan, H. F. Harvey.

"The Advantages of the Pyrometer for Obtaining Exact Results in Baking Porcelain," Weston A. Price, Cleveland. Discussion, L. E. Custer, H. M. Semans.

"Removable and Fixed Bridge-Work," Fred A. Peeso, Philadelphia, Pa. Discussion, Geo. H. Wilson, W. O. Hulick.

"Selected," Edward K. Wedelstaedt, St. Paul, Minn. Discussion, O. N. Heise, L. L. Barber.

"Pathological Irregularities," M. H. Fletcher, Cincinnati. Discussion, C. A. Hawley, W. S. Locke.

"Development and Maldevelopment Incident to Infant Dentition" (stereopticon), S. D. Ruggles, Portsmouth. Discussion, H. A. Smith, E. H. Raffensperger.

CLINICS—WEDNESDAY.

1. A. C. Searl, Owatonna, Minn., "Gold Filling, Mesio-Occlusal Surface of Molar or Bicuspid."
2. Henry C. Raymond, Detroit, Mich., "Porcelain Inlay."
3. G. W. Wasser, Cleveland, "High-Fusing Porcelain Inlay."
4. Frank J. Smith, Columbus, "Porcelain Inlay, Brewster's Body."
5. Edward E. Hall, Columbus, "Ambler's Tin and Sibley's Felt Gold."
6. H. C. Brown, Columbus, "A Method of reducing Pain in Cavity Preparation."
7. Chas. K. Teter, Cleveland, "Extracting under Prolonged Anæsthesia with Nitrous Oxide and Oxygen."
8. L. O. Green, Chicago, Ill., "Extracting under Local Anæsthesia."
9. C. H. Thompson, Athens, "The Use of Distilled Water for the Painless Extraction of Teeth."
10. M. E. Fenton, Cleveland, "Ethyl Chloride in Dental Surgery."
11. C. V. Lanum, Washington C. H., "Immediate Regulating."
12. Fred A. Peeso, Philadelphia, Pa., "Removable and Fixed Bridge-Work."
13. W. O. Hulick, Cincinnati, "Carved Cusps for Gold Crowns and Bridges."
14. Edward B. Spalding, Detroit, Mich., "A Shell of Porcelain reproducing the Entire Natural Enamel and thoroughly protecting the dentine of a Living Tooth when made necessary by Erosion or other Cause."

15. Geo. E. Bratton, Manchester, "One Method of making the Gold Bicuspid and Molar Crown."

16. E. S. Fuller, Piqua, "Partial Gold and Rubber Plates."

17. Weston A. Price, Cleveland, "The Advantages of the Pyrometer."

18. W. H. Hayden, Youngstown, "The Whiteside Crown as a Bridge Dummy."

19. Geo. H. Wilson, Cleveland, "Expansion and Compressibility of Plaster."

20. Varney E. Barnes, Cleveland, "Adjustable Regulating Appliances."

21. W. L. Gares, Columbus, "Method of repairing and packing Partial Dentures." (*b*) "Method of setting Logan Crown."

22. W. I. Jones, Nelsonville, "One Way to set a Fractured Inferior Maxillary."

23. Clare Smith, Columbus, "Crown- and Bridge-Work, Removable Facings."

24. R. C. Brophy, Chicago, Ill., "Porcelain Work, using Gasoline and Gas Furnaces."

25. F. W. Stephan, Chicago, Ill., "Anatomical Articulator."

26. L. E. Custer, Dayton, "Demonstration of a New Electric Appliance."

CLINICS—THURSDAY.

1. A. C. Searl, Owatonna, Minn., "Gold Filling, Mesio-Occlusal Surface of Molar or Bicuspid."

2. Geo. H. Woodbury, Cleveland, "High-Fusing Porcelain Inlays."

3. Gillette Hayden, Columbus, "Treatment of Diseased Pulps."

4. G. S. Junkerman, Cincinnati, "Soft Foil *versus* Cruelty."

5. W. G. Hamm, Chillicothe, "Pressure Anæsthesia, Immediate Pulp Extirpation."

6. J. W. McDill, Cleveland, "Flexible Rubber Attachment for Partial Rubber Dentures."

7. Holston Bartilson, Columbus, "A Practical Bridge, using Steele's Detachable Facing."

8. Sidney A. Rauh, Cincinnati, "Use of Napkins in Operative Work."

9. S. M. Weaver, Cleveland, "Pressure Anæsthesia by Automatic High-Pressure Syringe."

10. Karl C. Brashear, Columbus, "Anæsthesia, using Somnoform."

11. Nelson D. Edmonds, Wilmington, "Hollow Gold Inlays, Dr. C. N. Thompson's Method."

12. Fred A. Peeso, Philadelphia, "Removable and Fixed Bridge-Work."

13. H. E. Jenkins, Ironton, "Porcelain Jacket Crown."

14. E. Ballard Lodge, Cleveland, "Stereoscopic Dental Skiagraphs."

15. A. P. Bell, Zanesville, "Richmond Crowns."

16. J. O. Hawkins, Wellston, "Some Original Ideas on swaging Plates and Crowns."

17. Alden Bush, Columbus, "Anatomical Models for teaching Cavity Preparation, Dental Anatomy, etc."

18. J. C. Newton, Cleveland, "Knapp Crown."

19. M. L. Leob, Cleveland, "Crown and Bridge, using Over-Top Facing."

20. Edward B. Spalding, Detroit, Mich., "A Shell of Porcelain reproducing the Entire Natural Enamel and thoroughly protecting the Dentine of a Living Tooth when made necessary by Erosion or other Cause."

21. F. M. Casto, Cleveland, "Construction of Plain Bands (Orthodontia)."

22. H. H. Phillips, Cincinnati, "The Justi Porcelain Crown."

23. J. R. Bell, Cleveland, "Matrices."

24. R. C. Brophy, Chicago, Ill., "Cast-Metal Dentures."

25. M. H. Fletcher, Cincinnati, "Pathological Irregularities."

26. C. G. Myers, Cleveland, "Obtunding Sensitive Dentine."

---

### CALIFORNIA BOARD OF DENTAL EXAMINERS.

THE Board of Dental Examiners of California will hold an examination in San Francisco, commencing on Thursday, December 15, 1904.

F. G. BAIRD,
*Secretary.*

